# Patterns of Comorbidity in Hepatocellular Carcinoma: A Network Perspective

**DOI:** 10.3390/ijerph17093108

**Published:** 2020-04-29

**Authors:** Xiao-Min Mu, Wei Wang, Yu-Ying Jiang, Jia Feng

**Affiliations:** 1 Department of Medical Informatics, School of Public Health, Jilin University, Changchun 130021, China; muxm18@mails.jlu.edu.cn (X.-M.M.); w_w@jlu.edu.cn (W.W.); jiangyy_7@sina.com (Y.-Y.J.); 2 Cancer Systems Biology Center, Jilin University, Changchun 130033, China; 3 College of Computer Science and Technology, Jilin University, Changchun 130012, China

**Keywords:** hepatocellular carcinoma, comorbidity, correlation analysis, community detection

## Abstract

Hepatocellular carcinoma (HCC) is a common and fatal cancer. People with HCC report higher odds of comorbidity compared with people without HCC. To explore the association between HCC and medical comorbidity, we used routinely collected clinical data and applied a network perspective. In the network perspective, we used correlation analysis and community detection tests that described direct relationships among comorbidities. We collected 14,891 patients with HCC living in Jilin Province, China, between 2016 and 2018. Cirrhosis was the most common comorbidity of HCC. Hypertension and renal cysts were more common in male patients, while chronic viral hepatitis C, hypersplenism, hypoproteinemia, anemia and coronary heart disease were more common in female patients. The proportion of chronic diseases in comorbidities increased with age. The main comorbidity patterns of HCC were: HCC, cirrhosis, chronic viral hepatitis B, portal hypertension, ascites and other common complications of cirrhosis; HCC, hypertension, diabetes mellitus, coronary heart disease and cerebral infarction; and HCC, hypoproteinemia, electrolyte disorders, gastrointestinal hemorrhage and hemorrhagic anemia. Our findings provide comprehensive information on comorbidity patterns of HCC, which may be used for the prevention and management of liver cancer.

## 1. Introduction

Patients with cancer often carry the dual burden of the cancer itself and other coexisting chronic conditions [1]. Patients with liver cancer have a higher proportion of multiple medical conditions, leading to liver cancer being a major cause of premature illness and death [2,3,4]. Individuals suffering from multiple medical conditions are referred to as having comorbidity [5,6]. Comorbidity potentially affects the stages of the cancer journey from diagnosis, through treatment, to outcomes [7]. Patients with comorbidities are substantially more likely to experience complicated treatment, increased cost of care, decreased quality of life and lower survival probabilities than those without comorbidity [8,9]. In China, hepatocellular carcinoma (HCC) accounts for 90% of primary liver cancer. Therefore, understanding the patterns of diseases that coexist with HCC is important for disease screening and management.

While patients with HCC are at increased risk of comorbidity, few data sources are available for evaluating the comorbidity patterns among patients with HCC. Most studies of HCC comorbidity have focused on the relationship and mechanism of coexistence between HCC and other specific diseases, such as hepatitis B virus infection [10], hepatitis C virus infection [11,12] and type 2 diabetes [13,14], or on the epidemiological and clinical aspects of special populations with HCC, such as patients with obesity and elderly patients [15]. Although some comorbidities such as chronic infection with hepatitis B virus or hepatitis C virus, and type 2 diabetes are generally well recognized [10,16], many others may remain undiagnosed and can be detected by clinical data. Therefore, there is a need for comprehensive information from large clinical databases to identify the prevalence of liver cancer-related comorbidities and the comorbidity pattern of HCC.

Investigating network structures to understand the relationship between diseases is a recent research area in clinical science and medical informatics [17,18]. Network analysis is an efficient approach to analyze and visualize complex networks of diseases through identifying highly connected individual nodes and specific communities of nodes called modules [19]. Thus, the network perspective provides a straightforward overview of the co-occurrence of multiple disorders in a network structure and how they may interact and overlap [20]. The approach can be used to mine the relationships of multiple comorbidities, which can confirm existing knowledge regarding disease comorbidity as well as discover previously unappreciated associations among diseases.

In this study, we used the clinical data, including diagnostic data, derived from hospitals in Northeast China, to quantify the risk of liver cancer-related comorbidities. We established an HCC comorbidity network to obtain correlations between comorbidities by network measurements and discovered the pattern of disease comorbidity through the community detection method. We hope this study will be helpful to understand and manage diseases better in clinical settings.

## 2. Materials and Methods 

### 2.1. Data Source and Preprocessing

The data were derived from electronic medical records of 16 tertiary hospitals in Jilin Province, China during 2016–2018. The data contained more than 2 million records, including demographic data (such as age and sex), diagnostic data and medication data. The diagnostic data consisted of one primary diagnosis and up to 15 secondary diagnoses, which were coded by trained coders with the 10th revision of the International Classification of Diseases [21]. We used demographic and diagnostic data to analyze the comorbidity pattern of HCC. Data collected at the initial visit was used for this analysis. Inclusion criteria: data for patients diagnosed with hepatocellular carcinoma by clinical diagnosis, pathological diagnosis, etc., according to the Chinese practice guidelines for diagnosis and treatment of primary hepatic carcinoma. A total of 14,891 patients were included in the analysis. Ethical approval to conduct this study was obtained from the Ethics Committee of the School of Public Health, Jilin University, Changchun, China (grant number: ethical review 2020-02-01).

### 2.2. Statistical Analysis

The differences among the groups were compared by *χ*^2^ test or continuity correction, or Fisher’s exact test for categorical variables. All *p* < 0.05 from two-sided tests were accepted as significant. We used relative risk to measure the correlations between disease pairs [22]. For specific diseases A and B, relative risk was calculated according to the ratio of the observed prevalence of the disease pair to the expected prevalence [23]. The expected prevalence of the disease pair was computed as (prevalence of disease A) × (prevalence of disease B) [24]. A correlation was considered significant with relative risk > 1.0.

### 2.3. Network Analysis

We constructed the HCC comorbidity network with disease pairs whose correlations were significant. The weights of the disease pairs of which the two diseases co-occurred frequently would be large. Visualization of comorbidity networks can intuitively display the disease associations. We used Gephi 0.9.2 (WebAtlas, Paris, France), which is an open source software, for exploring and manipulating networks [25], to visualize the comorbidity network graphics. Each comorbidity was represented in the graph by a specific node and the diameter and color of nodes showed the prevalence and degree of the comorbidity, respectively. Diseases with larger degrees had more relationships with other diseases in the network. Links between nodes represented significant associations. The edges’ thickness represented the strengths of their association.

The community detection method was used for discovering the patterns of disease comorbidities. We adopted a computational algorithm proposed by Blondel [26] included in the Gephi to detect the highly interlinked topological clusters in the network. This method was based on modularity optimization. Modularity was the fraction of the edges that fell within the given groups of nodes minus the expected such fraction if edges were distributed at random. It was superior to other community detection methods in terms of computation time. In addition, the quality of the communities detected was good.

## 3. Results

### 3.1. Patient Characteristics

Table 1 presents the demographic characteristics of the HCC group, which comprised 14,891 patients. The HCC cohort was predominantly represented by seniors, with a mean age of 60 ± 11 years. To account for the distribution of age, the study group was stratified by age group, as shown in Table 1. The largest age group was 60–69 years (5251, 35.26%). There was a marked difference in HCC, which was more common in men, with 11,319 incident cases compared with 3572 in women. HCC occurred more often in men than in women, which agrees with the previous observation [4].

### 3.2. Distribution of Comorbidities

**Figure 1:**
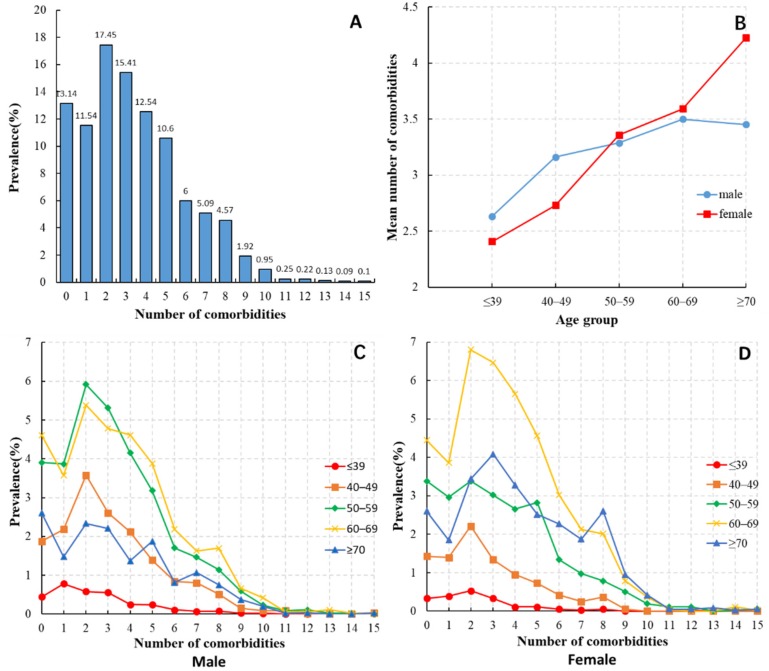
(**A**) Distribution of the number of comorbidities in patients with HCC. (**B**) Average number of comorbidities of HCC patients with different age and sex. (**C**) Distribution of the number of comorbidities of male HCC patients in each age group. (**D**) Distribution of the number of comorbidities of female HCC patients in each age group.

In terms of the number of comorbidities (Figure 1A), 13.14% of patients had only HCC; 11.54% had one comorbidity, 17.45% had two, 15.41% had three, 12.54% had four, 10.6% had five, and 19.32% had six or more. The main population of HCC patients had two comorbidities. As the number of comorbidities increased, the number of patients gradually decreased.

Young people had the fewest comorbidities, and the older the age, the greater the number of comorbidities (Figure 1B–D). In patients younger than 50 years, the number of comorbidities was higher in male than in female patients. In contrast, female patients had more comorbidities than male patients at age ≥ 70 years. The distribution curves for the number of comorbidities between male and female patients aged < 50 years were similar, but the prevalence of comorbidity among male patients was generally higher than that of female patients. The distribution curves of the number of comorbidities in male patients aged 50–59 and 60–69 years were basically the same as those of the female patients aged 60–69 years, and most had two comorbidities. Most female patients aged 50–59 years had up to five comorbidities. Patients aged ≥ 70 years had the most complicated comorbidity curve, with male patients often having up to five comorbidities and female patients up to eight.

### 3.3. Diseases Frequently Accompanying HCC

**Figure 2:**
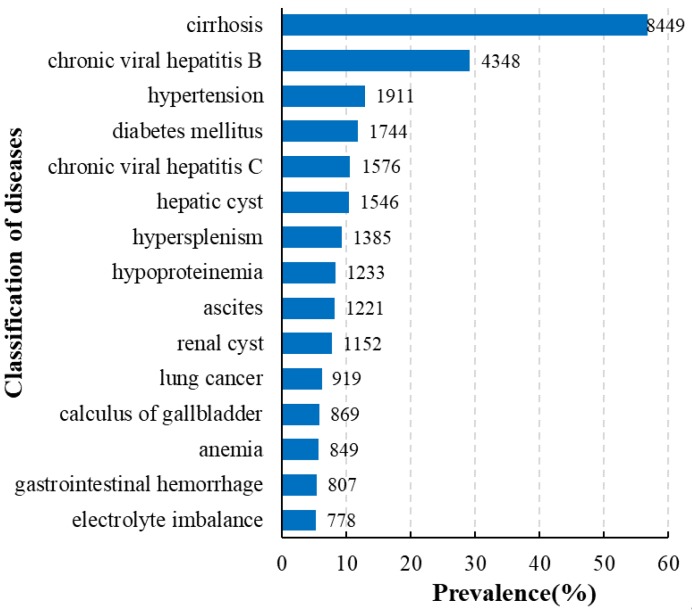
High-frequency comorbidities in patients with HCC.

Diseases that frequently accompany HCC are summarized in Figure 2. The five most frequent comorbidities were cirrhosis (8449, 56.74%), followed by chronic viral hepatitis B (4348, 29.20%), hypertension (1911, 12.83%), diabetes mellitus (1744, 11.71%), and chronic viral hepatitis C (1576, 10.58%).

The rankings for the top 15 most frequent comorbidities differed by sex (Table 2). The types of common comorbidities in male and female patients were similar. However, significant differences were observed between male and female patients regarding frequency of cirrhosis, chronic viral hepatitis B, hypertension, diabetes mellitus, hypersplenism, ascites, renal cysts, chronic viral hepatitis C, hypoproteinemia, anemia and coronary heart disease.

Table 3 shows prevalence of comorbidities by age group. The most common comorbidities in patients aged < 70 years were cirrhosis and chronic viral hepatitis B, while the most common comorbidities in patients aged ≥ 70 years were cirrhosis and chronic viral hepatitis C. In addition, some diseases could be considered as major comorbidities for specific age groups, for example, bone neoplasm and lymphoma for patients aged ≤ 39 years, and hypertension, cerebral infarction, coronary heart disease and heart failure for patients aged ≥ 60 years.

### 3.4. Comorbidities Network

**Figure 3:**
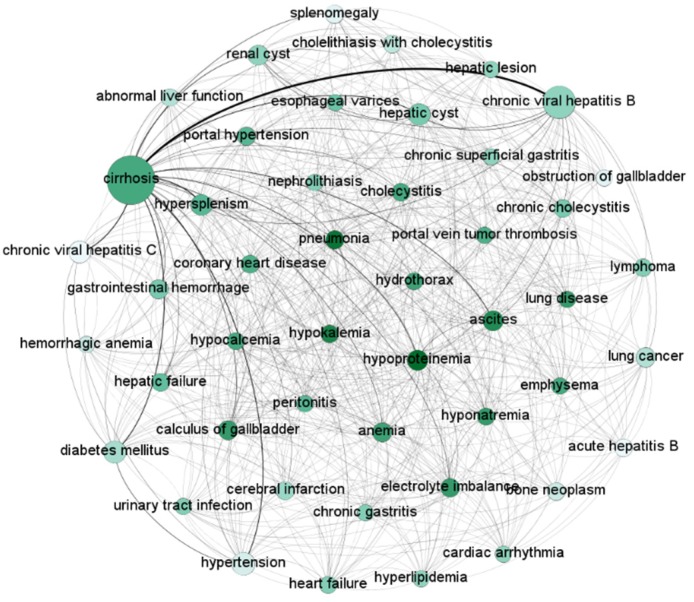
The HCC comorbidities network.

We constructed the HCC comorbidity network with diseases with prevalence > 1% and relative risk > 1.0 (Figure 3). The diameter and color of nodes shows the prevalence and degree of the comorbidity, respectively. Larger nodes represent higher prevalence and darker circles represent higher degrees. The HCC comorbidity network comprised 47 nodes representing each comorbidity and a total of 670 links representing those correlations. The closest link in the network was cirrhosis and chronic viral hepatitis B with 3258 co-occurrences. Metabolic disorders had more relationships with other diseases in the network, such as hypoproteinemia, hypokalemia and hyponatremia. Cirrhosis and metabolic disorders were at the hub of the network.

### 3.5. Network-based Clustering: Comorbidities Modules

**Figure 4:**
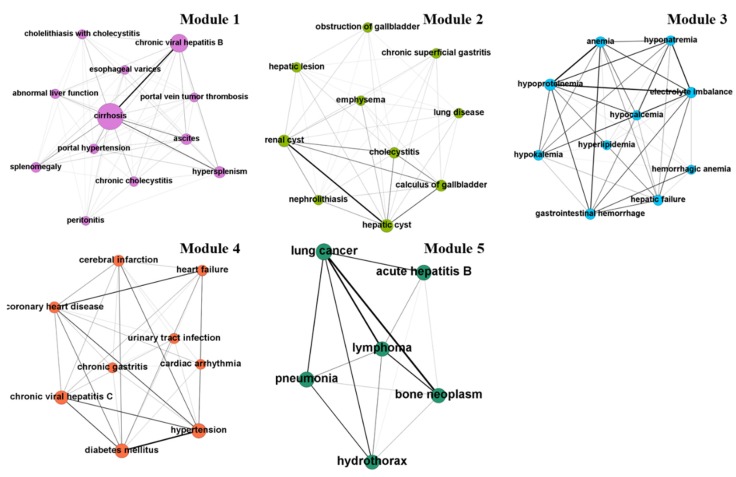
The HCC comorbidity modules.

The network contained distinct clusters or modules of highly interlinked nodes and the visual representation revealed a central theme (Figure 4). We enumerated each module rather than provide a name to avoid taxonomic bias. Module 1 comprised 12 nodes containing cirrhosis, chronic viral hepatitis B, portal hypertension, hypersplenism, ascites, peritonitis and other diseases caused by abnormal liver function. Module 2 comprised 10 nodes resulting from hepatic cyst, renal cyst, nephrolithiasis, gallbladder diseases and lung disease. Module 3 comprised ten nodes around metabolic disorders (e.g., hypoproteinemia, electrolyte imbalance and hypokalemia) and gastrointestinal hemorrhage. Module 4 comprised nine nodes in which comorbidities clustered around chronic disease (e.g., hypertension, diabetes mellitus, cerebral infarction and heart failure). Module 5 comprised six nodes around cancer (including lung cancer, lymphoma and bone neoplasm).

## 4. Discussion

We used network analysis to study common comorbidities in patients with HCC and the integrated relationship among different comorbidities, revealing high-impact comorbidities and patterns with meaningful clustering of diseases. These influential comorbidities could be targeted for specific intervention or for screening.

The results showed a strong association between HCC and cirrhosis and chronic viral hepatitis B and C. This finding supports those of previous studies, which have indicated that cirrhosis and chronic viral hepatitis B and C are strong risk factors for HCC [27,28,29]. Cirrhosis is the most important risk factor for HCC [30]. The most common causes for HCC include chronic viral hepatitis B and C infection [4]. HCC and cirrhosis had the highest association, and produced the following comorbidity pattern: HCC, cirrhosis, chronic viral hepatitis B, portal hypertension, ascites, hypersplenism, peritonitis and other common complications of cirrhosis. Previous comorbidity studies on cirrhosis revealed that portal hypertension, ascites and other above diseases are common comorbidities of cirrhosis [31,32]. Compared with the literature [33,34], our study reports a high rate of HCC without associated cirrhosis. There are probably three main reasons for this. The first reason is that there may be a variability in the methods used to monitor/diagnose fibrosis between different hospital centers. The second reason, which may be due to ethnicity, is that 20% of HCC patients do not have a background of cirrhosis in China [35]. The third reason is the time of data collection—the data comes from the first visit of patients with hepatocellular carcinoma, rather than from the entire stage of HCC. Alcoholic liver disease (ALD) and nonalcoholic fatty liver disease (NALFD) are high risk factors for cirrhosis [36]. Possibly because of the time of data collection, most ALD and NAFLD have been developed into cirrhosis. It is difficult to determine whether cirrhosis is caused by NAFLD, because with the increase of fibrosis, liver fat decreases. Even if a pathological biopsy is performed, the characteristic changes of NAFLD are not obvious. HCC is frequently accompanied by one or more components of metabolic disorders such as hypertension, diabetes mellitus, hypoproteinemia, electrolyte imbalance and hyperlipidemia. Metabolism is the most important function of the liver. The metabolism of sugar, protein, fat, vitamins and electrolytes is closely related to the liver. Metabolic disorder is associated with an increased risk of HCC [37,38]. Insulin resistance, glucose and lipid metabolic disorders and an abnormal release of inflammatory mediators are the common bases of HCC and diabetes mellitus [39]. Moreover, the results of the present study link HCC with metabolic dysfunction, gastrointestinal hemorrhage, coronary heart disease and cerebral infarction. Specifically, the association produced the following comorbidity patterns: HCC, hypertension with diabetes mellitus, coronary heart disease and cerebral infarction; and HCC with hypoproteinemia, electrolyte disorders, gastrointestinal hemorrhage and hemorrhagic anemia. Patients with HCC often suffer from severe cirrhosis, resulting in insufficient synthesis of coagulation factors, and as a result, HCC is often complicated with gastrointestinal bleeding and anemia [40]. However, the mechanism of relationship between electrolyte disorders and gastrointestinal bleeding is still unclear, which needs further experimental analysis.

Our study also found that different sexes and different age groups showed different comorbidity bias. With increasing age, patients had a significantly higher risk of comorbidities. Cirrhosis, chronic viral hepatitis B, hypertension and renal cysts were more common in male patients, while chronic viral hepatitis C, hypersplenism, hypoproteinemia, anemia and coronary heart disease were more common in female patients. The proportion of chronic diseases in comorbidities increases with age, such as hypertension, diabetes mellitus, coronary heart disease and cerebral infarction. This shows that elderly patients face a higher risk of comorbidity of chronic diseases and lower quality of life. It is suggested that elderly patients should pay more attention to their chronic disease comorbidities and strengthen prevention and management of comorbidities.

This study had several limitations. First, the data came from medical institutions in Northeast China, so the results were regional. Second, the data lack information about lifestyle factors such as diet, physical activity and medication, which may have influenced the results. Finally, this study was limited in determining precedence or causality of disease. On the basis of this study, we will further study the comorbidities of liver cancer and explore the relationship between comorbidities of liver cancer and patient physical status such as body mass index and alcohol consumption, to make a more detailed classification of different populations. Furthermore, we will attempt to analyze the differences of comorbidities in different stages and different types of liver cancer and incorporate comorbidity information into the staging system to predict the prognosis results.

## 5. Conclusions

This study explored the comorbidity patterns of HCC using network analysis and found that patients in different age groups and sexes also had significant differences in comorbidity risk. Cirrhosis is the comorbidity with the highest risk of HCC. The main comorbidity patterns of HCC were: HCC, cirrhosis, chronic viral hepatitis B, portal hypertension, ascites and other common complications of cirrhosis; HCC, hypertension, diabetes mellitus, coronary heart disease and cerebral infarction; and HCC, hypoproteinemia, electrolyte disorders, gastrointestinal hemorrhage and hemorrhagic anemia. These results can provide a reference for the clinical diagnosis and active prevention of HCC comorbidity and play a positive role in improving the quality of life of patients with HCC comorbidity.

## Figures and Tables

**Table 1:** Demographic characteristics of patients with hepatocellular carcinoma (HCC).

Variables	Patients with HCC (*n* = 14,891) *n* (%)
Age, year	
≤ 39	431 (2.89)
40–49	2172 (14.59)
50–59	4388 (29.47)
60–69	5251 (35.26)
≥70	2649 (17.79)
Sex	
Female	3572 (23.99)
Male	11,319 (76.01)
Nationality	
Han	13,161 (88.38)
Korean	1697 (11.40)
Man	117 (0.79)
Other	84 (0.56)
Marital status	
Married	13,987 (93.93)
Single	904 (6.07)

**Table 2:** Comorbidity prevalence and comparison between male and female patients with HCC.

Disease	Male Patients (*n* = 11,319)		Female Patients (*n* = 3572)		*p*-Value
	*n* (%)	Rank	*n* (%)	Rank	
Cirrhosis	6495 (57.38)	1	1954 (54.70)	1	0.0049
Chronic viral hepatitis B	3557 (31.43)	2	791 (22.14)	2	<0.001
Hypertension	1389 (17.02)	3	522 (14.61)	4	<0.001
Diabetes mellitus	1365 (11.97)	4	379 (10.61)	6	0.0189
Hepatic cyst	1179 (10.42)	5	367 (10.27)	7	0.809
Hypersplenism	989 (10.20)	6	396 (11.09)	5	<0.001
Ascites	971 (8.74)	7	250 (7.0)	10	0.0027
Renal cyst	961 (8.58)	8	191 (5.35)	17	<0.001
Chronic viral hepatitis C	941 (8.49)	9	635 (17.78)	3	<0.001
Hypoproteinemia	882 (8.31)	10	351 (9.83)	8	<0.001
Lung cancer	700 (7.79)	11	219 (6.13)	12	0.908
Gallbladder stones	666 (5.88)	12	203 (5.68)	13	0.655
Gastrointestinal hemorrhage	614 (5.42)	13	193 (5.40)	15	0.961
Anemia	599 (5.39)	14	250 (7.00)	9	<0.001
Electrolyte imbalance	580 (5.12)	15	198 (5.54)	14	0.327
Coronary heart disease	393 (3.47)	25	239 (6.69)	11	<0.001

**Table 3:** Prevalence of comorbidities by age category (years).

Disease	≤39 (*n* = 431) *n* (%)	40–49 (*n* = 2172) *n* (%)	50–59 (*n* = 4388) *n* (%)	60–69 (*n* = 5251) *n* (%)	≥70 (*n* = 2649) *n* (%)	*p*-Value
Cirrhosis	206 (47.80)	1368 (62.98)	2654 (60.48)	3008 (57.28)	1213 (45.79)	<0.001
Chronic viral hepatitis B	139 (32.25)	895 (41.21)	1619 (36.90)	1351 (25.73)	344 (12.99)	<0.001
Hypertension	2 (0.46)	161 (7.41)	408 (9.30)	841 (16.02)	499 (18.84)	<0.001
Diabetes mellitus	3 (0.70)	160 (7.37)	571 (13.01)	702 (13.37)	308 (11.63)	<0.001
Chronic viral hepatitis C	1 (0.23)	36 (1.66)	267 (6.08)	738 (14.05)	534 (20.16)	<0.001
Hepatic cyst	24 (5.57)	157 (7.23)	460 (10.48)	642 (12.23)	263 (9.93)	<0.001
Hypersplenism	37 (8.58)	231 (10.64)	401 (9.14)	523 (9.96)	193 (7.29)	<0.001
Hypoproteinemia	20 (4.64)	189 (8.70)	326 (7.43)	413 (7.87)	285 (10.76)	<0.001
Ascites	35 (8.12)	171 (7.873)	418 (9.53)	393 (7.48)	204 (7.70)	0.005
Renal cyst	12 (2.78)	125 (5.76)	347 (7.91)	443 (8.44)	225 (8.49)	<0.001
Lung cancer	66 (15.31)	149 (6.86)	246 (5.61)	316 (6.02)	142(5.36)	<0.001
Gallbladder stones	9 (2.09)	134 (6.17)	217 (4.95)	337 (6.42)	172 (6.49)	<0.001
Anemia	11 (2.55)	118 (5.43)	243 (5.54)	285 (5.43)	192 (7.25)	<0.001
Gastrointestinal hemorrhage	21 (4.87)	121 (5.57)	248 (5.65)	261 (4.97)	156 (5.89)	0.404
Electrolyte imbalance	17 (3.94)	144 (6.63)	197 (4.49)	266 (5.07)	154 (5.81)	0.002
Bone neoplasm	25 (5.80)	74 (3.41)	151 (3.44)	172 (3.28)	62 (2.34)	0.002
Lymphoma	24 (5.57)	68 (3.13)	162 (3.69)	197 (3.75)	57 (2.15)	<0.001
Splenomegaly	18 (4.18)	135 (6.22)	231 (5.26)	242 (4.61)	78 (2.94)	<0.001
Cerebral infarction	NA	8 (0.37)	83 (1.89)	300 (5.71)	226 (8.53)	<0.001
Coronary heart disease	2 (0.46)	9 (0.41)	94 (2.14)	260 (4.95)	267 (10.08)	< 0.001
Heart failure	NA	2 (0.09)	58 (1.32)	144 (2.74)	175 (6.61)	<0.001

^1^ NA = not applicable.
